# CRISPR/Cas9 nanoeditor of double knockout large fragments of E6 and E7 oncogenes for reversing drugs resistance in cervical cancer

**DOI:** 10.1186/s12951-021-00970-w

**Published:** 2021-08-05

**Authors:** Xianhuang Li, Mingming Guo, Bei Hou, Bin Zheng, Zhiyun Wang, Mengqian Huang, Yanan Xu, Jin Chang, Tao Wang

**Affiliations:** 1 School of Life Sciences, Tianjin University, 92 Weijin Road, Nankai, 300072 Tianjin, China; 2 Academy of Medical Engineering and Translational Medicine, Tianjin Key Laboratory of Brain Science and Neural Engineering, Xincheng Hospital of Tianjin University, Tianjin University, 92 Weijin Road, Nankai, 300072 Tianjin, China; 3 School of Environmental Science and Engineering, Tianjin University, 92 Weijin Road, Nankai, 300072 Tianjin, China

**Keywords:** Drug resistance, CRISPR/Cas9, Nanoeditor, Oncogene deletion, Cervical cancer

## Abstract

Drug resistance of tumor cells is always a headache problem in clinical treatment. In order to combat chemotherapy-resistance in cervical cancer and improve treatment effect, we design a CRISPR/Cas9 nanoeditor to knock out two key oncogenes E6 and E7 that lead to drug tolerance. Meanwhile, the deletion of these two oncogenes can effectively reactivate p53 and pRB signaling pathways that inhibit the growth of tumor cells. Our results demonstrated the nanoeditor could simultaneously delete two oncogenes, and the size of DNA fragments knocked out reaches an unprecedented 563 bp. After the preparation of cationic liposomes combined with chemotherapy drug docetaxel (DOC), this nanosystem can significantly inhibit the drug tolerance of cancer cells and improve the therapeutic effect of cervical cancer. Therefore, this study provides a promising strategy for the treatment of cervical cancer by combining chemotherapy and double-target gene therapy. This strategy can also be applied in other disease models to customize personalized anti-tumor strategies by simply changing chemotherapy drugs and targeted genes.

**Supplementary Information:**

The online version contains supplementary material available at 10.1186/s12951-021-00970-w.

**Figure:**
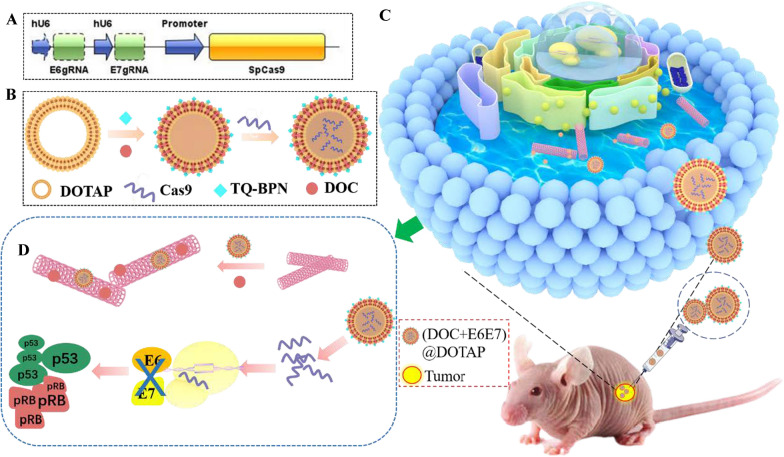

## Introduction

Chemotherapy is one of the most important strategies of cancer treatment [1]. However, chemo-resistance continues to be the principal limiting factor to achieving cures in patients with cancer [2]. Evidence shows that even when chemotherapy causes tumors remission quickly, while drug resistance would appear in the course of long-term drug use, which leads to the failure of tumor treatment [3–5]. Chemo-resistance is one of the key causal factors in cervical cancer death [6]. In clinical practice, it is necessary to increase the dosage of drugs to improve the effect of tumor treatment, with which toxic side effects often increases [7]. In order to overcome this problem, more and more researchers focus on developing novel anti-tumor drugs targeting new protein targets, or combining multiple drugs simultaneously to improve the therapeutic effect of cancer. However, the development of new drugs is time-consuming and expensive. Even developed new drug makes no evasion to the evolution of new resistance mechanism of cancer cells. Therefore, genetic modification related to drug resistance in cancer cells at soure may be the key to solve this problem.

The interaction between drug and tumor microenvironment is very complex which affects each other throughout. Property of cancer cells develops remarkable resistance to various treatments which target different molecular pathways [8–10]. Recent evidence suggests that p53 gene, are playing an important role in drug resistance of tumor cells [11]. P53 can use Y-BOX binding protein to down-regulate the expression of resistance-related proteins, thereby increase the sensitivity of tumor cells to chemical drugs [12]. Alterations in cellular uptake and efflux, increased detoxification, and CDK1 intervention affected by p53 status are associated with the sensitivity of cancer cells to docetaxel and the development of drug resistance [13]. Loss of p53 can impair apoptosis process, consequently promoting the formation of drug resistance, which is also a major feature of cancer cells [11, 14]. Sultana et al. [15] demonstrated that chemosensitivity was associated with p53-Bax-mediated apoptosis in cervical cancer. Genomic studies have revealed that the expression of anticancer genes pRB and p53 will be down-regulated or even lost in tumor cells after long-term treatment, resulting in drug resistance [16–18]. Cervical cancer cells with HPV-16 E7 expression or silenced for pRB acquire resistance to anti-proliferative effect of antineoplastic drugs [19]. Based on these findings, it is hoped that interfering expression of key oncogenes reverse the development of drug resistance in tumor cells. RAN interference technique is one of the most common methods to regulate target gene expression, e.g. inhibition of the expression of multiple drug resistance related genes by RNA interference improves the chemotherapy effect of cervical cancer [20, 21]. However, RNA has a short timeliness, and with the degradation of RNA, the expression of drug-resistant genes also resumes immediately [22]. Therefore, more stable and efficient genetic modification methods are urgently needed to reverse the drug resistance of cancer cells and enhance the chemotherapy effect of tumors.

We report a CRISPR/Cas9 vector system that enables efficient editing of two target sites of oncogene to permanently delete the DNA fragments between the target sites [23, 24]. The vast majority of cervical cancers are caused by human papilloma virus (HPV) infection, with two oncogenes, E6 and E7, of the virus integrated into host cells [25]. Therefore, overexpression of these two genes is the key to drive and sustain cervical cell cancerization [26]. Therefore, we speculated that these two important oncogenes were also related to the occurrence of drug resistance in tumor cells. The gene nanoeditor we have developed, by carrying two pairs of primers, can simultaneously and efficiently knock out two key oncogenes that maintain tumorigenesis in cervical cancer cells. After the oncogene was deleted, we found that the p53 and pRB signaling pathways which inhibit tumor cell growth were reactivated, which combined with docetaxel (DOC) and significantly improve the killing efficiency of cervical cancer cells. Because the E6 and E7 genes are from HPV, they are only found in cervical cancer cells, which ensures specificity and safety of this nanoscale editing system. To trace the nanosystem in real-time, a fluorescent molecule of TQ-BPN [27, 28], owning to the aggregation-induced emission (AIE) effect in the infrared region, was selectively embedded in the hydrophobic shell of the amphiphilic cationic liposome [29, 30]. The nanosystem formed by cationic liposome encapsulation negatively charged CRISPR/Cas9 plasmid and the hydrophobic DOC has shown excellent therapeutic efficacy and negligible side effects in a mouse model of cervical cancer [31]. Therefore, this study provides a promising strategy for the treatment of cervical cancer by combining chemotherapy and double-target gene therapy. This approach can also be applied in other disease models to customize personalized anti-tumor strategies by simply changing chemotherapy drugs and targeted genes.

## Materials and methods

### CRISPR/Cas9 plasmid constructs and sgRNA design

Two pairs of sgRNAs were designed by using the ZiFit Web application (http://zifit.partners.org/) to target the amino-terminal regions of the HPV-18 E6 and E7 open reading frames (ORFs) (Additional file 1: Fig. S1A). RNA-guided DNA endonucleases were constructed by cloning HPV-specific sgRNAs into the px330 vector (Addgene Catalog #42,230) expressing S. pyogenes Cas9. The plasmids px330-E6 and px330-E7 were constructed, using the following gene-specific sgRNA sequences, as outlined: HPV-18 E6(GGCGCTTTGAGGATCCAACA), HPV-18 E7(GGAGCAATTAAGCGACTCAG). U6-E7 sgrna was amplified from px330-E7 by PCR and inserted into px330-E6 to construct px330-E6E7 plasmid, termed E6E7.

### Preparation of DOTAP-AIEgen @DOC + E6E7 formulations

The negative-charged plasmid serves as a core and the cationic liposome serves as a shell. 1 mg of 1,2-dioleoyl-3-trimethylammonium-propane (chloride salt) (DOTAP), TQ-BPN and 0.5 mg of Docetaxel (DOC) were dissolved in chloroform and treated by thin film hydration (30℃, 100 rpm) on a rotary evaporator (Heidolph Hei-VAP Value Digital) and resuspended in PBS followed by an ultrasonic dispersion to form liposomes (final concentration of 10 mM). The 0.2 mg negative-charged plasmid was encapsulated by the cationic lipid shell by incubating the plasmid and the liposomes at room temperature for 15 min to yield (DOC + E6E7) @DOTAP. (DOC + E6E7) @DOTAP was dispersed in the Opti-MEM® medium for transfection experiments in vitro. For the in vivo transfection experiments, (DOC + E6E7) @DOTAP was dispersed in PBS before administration.

### Characterization

The shape and morphology of the samples were determined via transmission electron microscopy (TEM). The particle size and zeta potential of various nanoparticles were measured at 25 °C using dynamic light scattering (Malvern Zetasizer nano ZS, Malvern, UK). The fluorescence of the samples was investigated by EnSpire Multilabel Reader.

### In vitro transfection efficiency measurement

Cells were cultured in Dulbecco’s modified Eagle’s medium (DMEM), 10 % fetal bovine serum (FBS), 1 % penicillin-streptomycin mixture (all from Gibco, USA) and incubated at 37 °C with 5 % CO_2_. The cells were routinely harvested by 0.25 % Trypsin-EDTA (Gibco). Cells were seeded into 24-well plates (1 × 10^5^ cells/well) or 96-well plates (2 × 10^4^ cells/dish) (NEST) in 500 µL complete DMEM with 10 % FBS, followed by incubated at 37 °C for 12 h under 5 % CO_2_ atmosphere. Then, the culture medium was removed and the complex of nanocarriers-plasmid DNA was added. After incubation for 48 h, the culture media was removed, and the cells were washed for twice with cold PBS. Then, imaging was performed using an inverted Olympus fluorescence microscope (IX-51) or confocal microscopy (PerkinElmer, USA).

Meanwhile, adherent cells were harvested by adding M-PER lysis buffer, Halt protease and phosphatase inhibitor (Thermo Scientific). Non-adherent cells were harvested by centrifugation of cell media, and then the resulting cell pellet was combined with the cell lysate from corresponding adherent cells. Cell lysates were subjected to electrophoresis on 10 % SDS-PAGE and transferred to PVDF membranes (25 V, 20 min). Membranes were blocked for 1 h with 5 % skim milk in TBST (Tris-HCl, NaCl and tween20 solution), and then incubated with primary antibody 1:1000 diluted with 5 % skim milk overnight at 4 °C on a platform rocker. The membranes were then washed for 3 × 6 min each with TBST and incubated with anti-rabbit IgG–HRP secondary antibody at 1:2000 in 5 % skim milk for 1 h at room temperature. After 3 × 6 min-washing with TBST, the membranes were exposed to chemiluminescent substrate (BioRad Clarity) for 5 min and imaged with a CCD-camera on an Alpha Innotech FluorChem FC2 imaging station.

### Quantitative reverse transcription PCR (qRT-PCR)

The RNA was acquired from striatum with TRIzol reagent. Concentrations of RNA was acquired were then evaluated with Nano Drop 2000 and utilized to combine the complementary DNA with a RevertAid First Strand cDNA synthesis kit (Thermo Fisher Scientific, MA, USA). Next, qRT-PCR was performed with HPV18 E6, HPV18 E7, and Actin-specific primers on an Bio-rad,CFX96 touch real time system with gene PCR master mix (Thermo Fisher Scientific, MA, USA). Primers are listed below.

**Table:** 

HPV18 E6-F	CGCGCTTTGAGGATCCAACA
HPV18 E6-R	ACCTCTGTAAGTTCCAATACTGTCT
HPV18 E7-F	CGAGCAATTAAGCGACTCAG
HPV18 E7-R	GTTGTGGTTCGGCTCGTCGG
gRNA-R	CGACTCGGTGCCACTTTTTCAAGTTG

The relative mRNA expression of each group was compared utilizing the 2^−ΔΔCt^ method

### Cytotoxicity assay of different samples

The cytotoxicity of samples against cells was tested in vitro using 3-(4,5-dimethylthiazol-2-yl)-2, 15-diphenyltetrazolium bromide (MTT) assay. The cells were seeded into 96-well plates in 100 µL of DMEM with 10 % FBS, followed by incubation at 37 °C for 24 h under 5 % CO_2_. Then, 100 µL of medium containing requested amount of sample was added. The cells were incubated for another 24 h at 37 °C. Then, 20 µL of MTT solution was added to each well and incubation was continued for another 4 h. The culture medium was removed, and 200 µL of DMSO was added into each well to dissolve the formazan. The absorbance of each well was measured at 490 nm by a microplate reader (Model 680, Biorad). The survival percentage was then calculated as compared to that of untreated cells (100 % survival). In addition, after the cells transfection for 48 h, the cell viability was also evaluated by dyeing with 7-AAD for dead/late apoptosis cell visualization abided by manufacture’s suggestion (Invitrogen) and images were obtained using inverted fluorescence microscope (Olympus, IX-51).

### Flow cytometry (FCM)

The cells were seeded into 6-well plates at a density of 2 × 10^5^ cells per well and cultured for 24 h before the agent administration. After incubation with the complex of nanocarriers-plasmid DNA for 4 h, the culture media were replaced with fresh media and the cells were further cultured for 48 h. The cells were incubated with Annexin V-FITC and PI for 10 min and collected, fixed with 1 % paraformaldehyde, and examined with FCM (BD Biosciences). 1 × 10^4^ cells were analyzed per sample. The data were analyzed using FACSDiva v. 6.1.3 and FlowJo v. 7.6.2 software.

### Animals and tumor model building

Female BALB/c nude mice (5–6 weeks old) were purchased from Beijing HFK Bioscience Co., Ltd. (Beijing, China) and used in accordance with the guidelines of the Chinese National Science and Technology Committee. For tumor model building, 1 × 10^7^ HeLa cells suspended in 100 µL of PBS were subcutaneously injected into the right flanks of the mice. In addition, the in vivo therapeutic experiments were proceeded when the tumor volume reached about 100 mm^3^. Each mouse in each group was injected with 100 µL of PBS, DOC@DOTAP, E6E7@DOTAP and (DOC + E6E7) @DOTAP, and then the tumor growth status was recorded daily. The tumor volume was calculated as (tumor length) × (tumor width)^2^/2. The TQ-BPN images of mice were obtained at predetermined time using the ex/in vivo imaging system (Maestro, USA) with a 650 nm excitation wavelength. In addition, the histological analysis and statistical analysis were performed.

### Statistical analysis

All experiments are repeated at least three times. Data are presented as the means ± SD. A One-way ANOVA test was used to determine significance among groups. *p < 0.05 and **p < 0.01 were considered significant and highly significant, respectively.

## Results and discussion

### Construction of CRISPR/Cas9 plasmid to target the deletion of oncogenes

Cervical cancer is mainly caused by human papilloma virus (HPV) infection [32], and the overexpression of E6 and E7 oncogenes in HPV infected cervical cancer cells is the key to maintain the growth and proliferation of cancer cells [33, 34]. Therefore, knockout of E6 and E7 oncogenes is expected to inhibit the growth and spread of cervical cancer from the source. We designed a highly efficient CRISPR/Cas9 gene-editing system to target the E6 and E7 oncogenes of HPV that are missing and integrated into the genome of HeLa cells. The constructed sgRNAs were completely complementary to the 5–24 nucleotides in the open reading frame (ORF) of E6 gene and the 84–103 nucleotides in the ORF of E7 gene. These sgRNAs were expressed under the control of a U6 RNA polymerase III promoter present in the px330 expression vector, which assist the targeted excision of E6 and E7 oncogenes by Cas9 proteins (Additional file 1: Fig. S1).

**Fig. 1:**
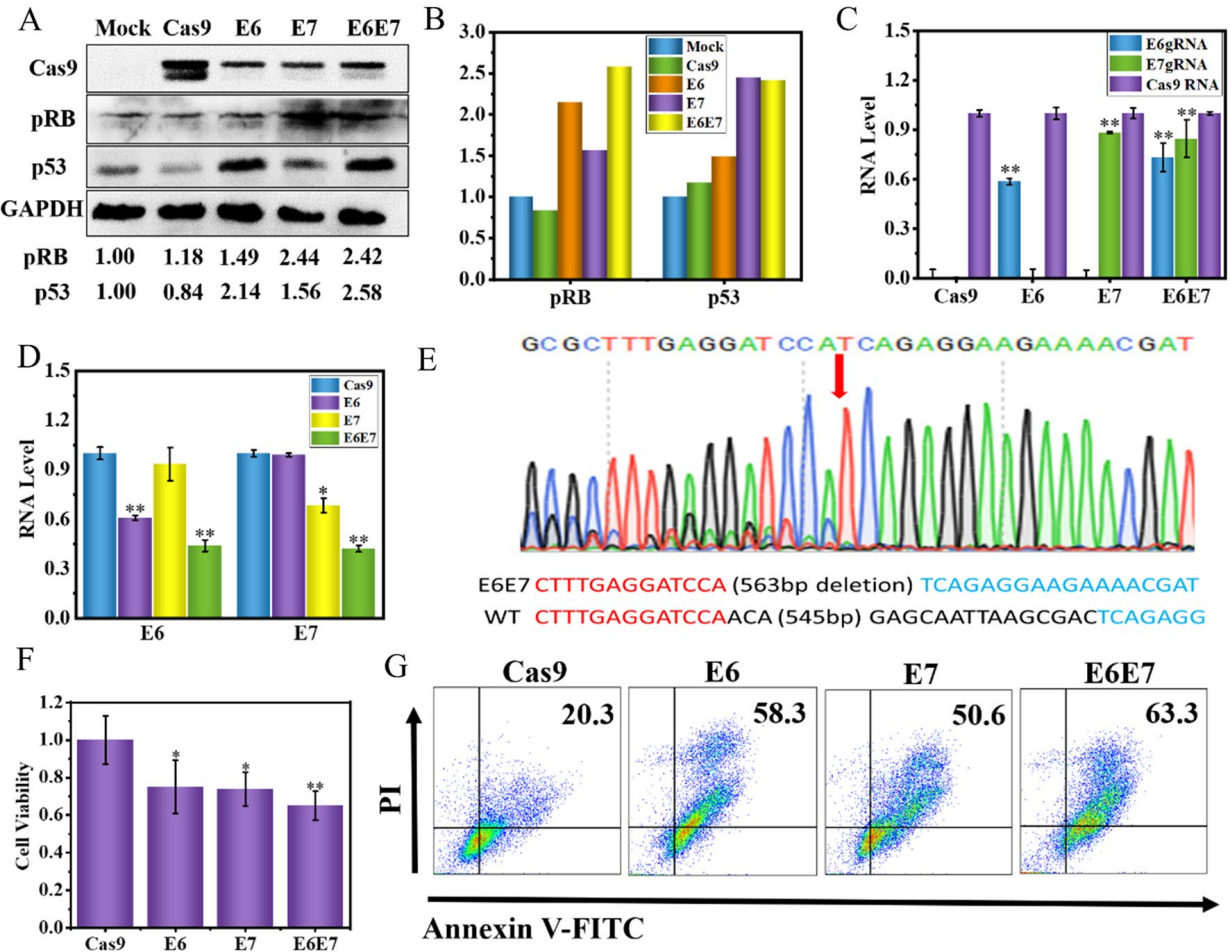
CRISPR/Cas editing of the HPV E6 and E7 genes reduces cell viability and induces key growth control proteins. **A** HeLa (HPV18 positive) cells were treated with target-specific gRNAs or control for 48 h before protein expression of various growth control proteins (p53 for E6 targeting, RB for E7 targeting) was determined by western blot analysis. **B** The relative density of bands was quantified using ImageJ software. **C** qRT–PCR Measurement of expression of sgRNA. *p < 0.05, **p < 0.01 vs. control. **D** E6 and E7 mRNA expression in the HeLa cells was decreased significantly after transfection for 48 h. *p < 0.05, **p < 0.01 vs. control. **E** Examples of direct sequencing of PCR products containing targeted sites. **F** and **G** CCK-8 assay and cell apoptosis assay. Cells were treated with target-specific gRNAs (E6, E7, or E6E7), transfected for 48 h before CCK-8 assay and cell apoptosis assay. *p < 0.05, **p < 0.01 vs. control

Both E6 and E7 expressions are known to be required for HeLa cell growth and survival, so we next wondered if targeting E6 and E7 with an RNA-guided DNA endonuclease would indeed inactivate E6 and E7 function. As noted above, the HPV E6 protein functions to repress the expression of the host p53 tumor suppressor so that a loss of E6 function is expected to result in the excitation of p53 expression. Targeting HPV E7 genes restored Rb protein expression. The p53 and pRB play an important role in chemoresistance. As shown in Fig. 1A, we have detected an increase in p53 and RB expression when Cas9 targets E6 and E7 gene respectively. Furthermore, quantitative analysis showed that the expression of Rb protein and p53 protein in E6E7 group increased by 2.58-fold and 2.42-fold respectively compared with mock group (Fig. 1A, B). We next used reverse transcription–quantitative PCR (qRT–PCR) to measure the expression of sgRNAs in HeLa cell with a two-target construct targeting the E6 and E7 genes (Fig. 1C). The expression level of E6sgRNA and E7sgRNA was the same as that of E6sgRNA or E7sgRNA, which indicates that cloning two sgRNA expression cassettes into the vectors does not reduce the expression level of sgRNA. However, the E6 and E7 mRNA expression levels in the HeLa cells was decreased significantly after transfection Cas9 plasmids, especially in the E6E7 group (Fig. 1D). Thus, this study obtained a single CRISPR/Cas9 vector here, termed E6E7. It was verified by Sanger sequencing that HPV18 E6 and E7 DNA in HeLa cells were deleted, with 563 bp, after the transfection (Fig. 1E and Additional file 1: Fig. S2).

To determine the overall efficiency of our gene therapy, we performed PCR and T7E1 assays. By PCR amplification of the target region, we found that about 55.2 % of the genes were deleted (Additional file 1: Fig. S3A). Further analysis of T7E1 showed that about 37.7 % of the undeleted genes were edited (Additional file 1: Fig. S3B). These results exhibit that our CRISPR/Cas9 system can successfully delete both E6 and E7, two crucial oncogenes to maintain the survival and growth of cancer cells, from the genome of HeLa cells.

### Inhibits cell growth and triggers cell apoptosis after oncogene deletion

Persistent infection with high-risk HPV, especially type 18, is responsible for 99.7 % of cervical cancer cases. After infection, HPV over-expression of the E6 and E7 oncogenes drive and sustain cervical cancer. The reactivation of p53 tumor suppressor pathway when targeting E6 oncogene, or the restoration of the tumor suppressor retinoblastoma protein (RB) pathway when targeting the E7 oncogene, which gave partly explanation of the cell death. To verify the effect of overexpression of p53 and pRB on cell growth and apoptosis, we performed CCK-8 assay and cell apoptosis assay. Simultaneous expression of two sgRNAs simultaneously up-regulates p53 and pRB expression compared to the expression of only one sgRNA, resulting in an inhibition of HeLa cell growth and viability by approximately 10 % (Fig. 1E). The co-expression of p53 and pRB up-regulates the counts of apoptosis by approximately 14–16 % (Fig. 1F). Therefore, the CRISPR/Cas9 system we designed up-regulated the expression of p53 and pRB genes after deleting the E6 and E7 genes of HeLa cells, which in turn caused HeLa cells apoptosis.

### CRISPR/Cas9 system specifically targets HeLa cells

**Fig. 2:**
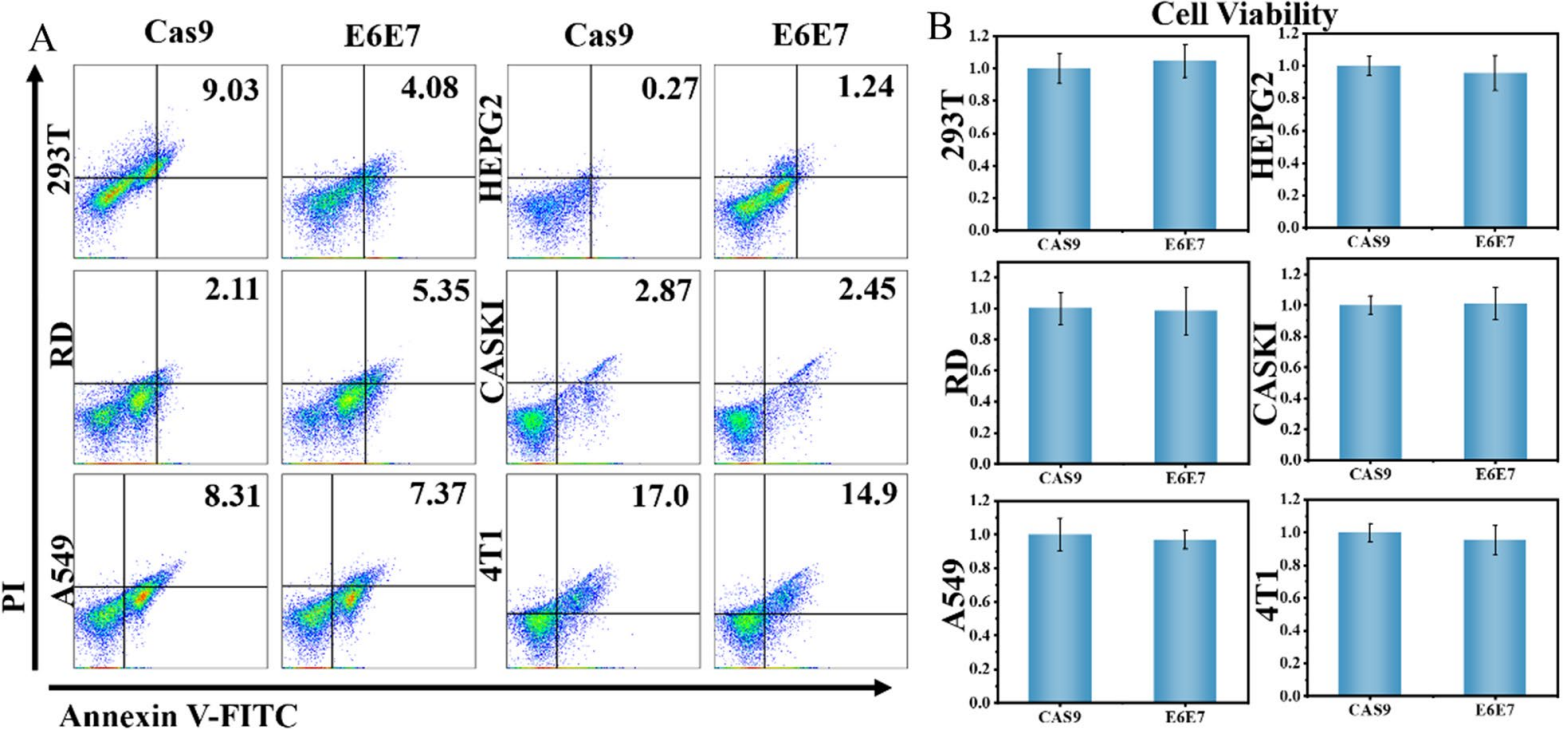
CRISPR/Cas9-based therapy is targeted. **A** and **B** CCK-8 assay and cell apoptosis assay. Cells (293T; RD; A549; HEPG2; CasKi, HPV16 positive; 4T1) were treated with target-specific gRNA (E6E7) or control gRNA (non-specific), for 48 h before CCK-8 assay and cell apoptosis assay

The CRISPR/Cas9 system is a gene therapy that specifically targets a certain gene. In Fig. 1, we verified that the CRISPR/Cas9 system reduces the expression of E6 and E7 genes in HeLa cells, thereby activating p53 and pRB to promote apoptosis. However, the specific targeting of the CRISPR/Cas9 system has the top priority for eliminating HeLa cells in the treatment of cervical cancer. Therefore, we verified the biosafety of the CRISPR/Cas9 system for other cells. We used human embryonic kidney 293T cells (293T), human rhabdomyosarcoma cells (RD), human lung carcinoma cells (A549), human liver hepatocellular carcinoma cells (HEPG2), HPV16 cervical cancer cells (Caski) and murine mammary carcinoma cells (4T1) by Cell Counting Kit-8 and flow cytometry to detect the specific targeting of the CRISPR/Cas9 system, which appears no activity effect of non-target cells by sgRNA (Fig. 2A, B). On-target specificity of gene editing, CRISPR/Cas9-based therapies to target organs with minimal systemic toxicities, and demonstrated lack of long-term adverse events. Calcein-AM/PI staining shows the same result (Additional file 1: Fig. S4). These results prove that the CRISPR/Cas9 system specifically targets HPV-18 positive cells and induces apoptosis without affecting the activity of other cells.

### Construction of CRISPR/Cas9 nanocarrier

**Fig. 3:**
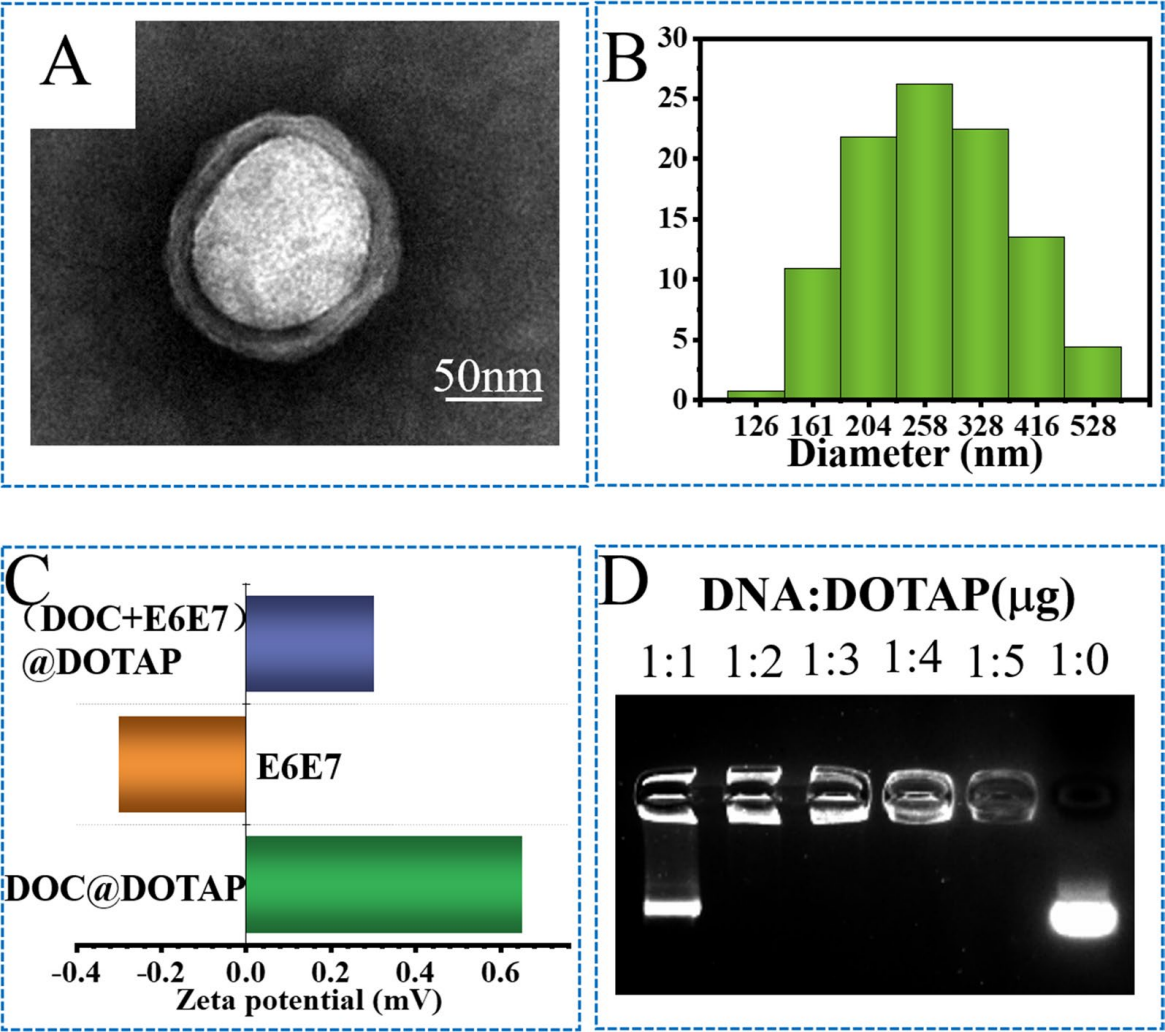
Preparation and characterization of nanosystem. **A** Transmission electronic microscopic image of (DOC + E6E7) @DOTAP. **B** DLS characterization of (DOC + E6E7) @DOTAP. **C** Zeta potential of DOC@DOTAP, E6E7 and (DOC + E6E7) @DOTAP. **D **Binding ability of DOTAP to CRISPR/Cas9 vectors at different ratios demonstrated by the agarose gel retardation assay

A major challenge for CRISPR/Cas9 therapies is the development of effective in vivo delivery platforms. At present, the most effective cervical cancer treatment is chemotherapy. However, the long-term use of chemotherapy drugs leads to the emergence of cell resistance, which is a major bottleneck in the treatment of cervical cancer. In order to reverse the resistance of transformation therapy and improve the treatment effect, we used CRISPR/Cas9 and chemotherapy drug Docetaxel (DOC) in combination and constructed a CRISPR/Cas9 nanosystem. In order to improve the uptake efficiency of cells, we use cationic liposome DOTAP to encapsulate the CRISPR/Cas9 system and the chemotherapy drug DOC to prepare nanoparticles. The CRISPR/Cas9 nanosystem was tested by transmission electron microscopy (TEM) and dynamic light scattering (DLS) to a diameter of approximately 200 nm (Fig. 3 A, B and Additional file 1: S5), whose size was controlled by agitation speed. Zeta potential shows that DOC@DOTAP is 0.65 mV, E6E7 is -0.3 mV, and the final potential of (DOC + E6E7) @DOTAP is 0.3 mV (Fig. 3C). We then investigated the co-assembly of DOTAP with negatively charged Cas9 DNA at different weight ratio. An electrophoretic mobility shift assay (EMSA) showed that DOTAP produced large retardation of DNA at weight ratio of 2 (Fig. 3D). The results indicate that the CRISPR/Cas9 nanosystem, which can be successfully used for in vivo delivery, has been successfully constructed and expected to be used for in vivo targeted therapy of cervical cancer.

### Fluorescence imaging and cellular uptake of the nanoparticles in vitro

**Fig. 4:**
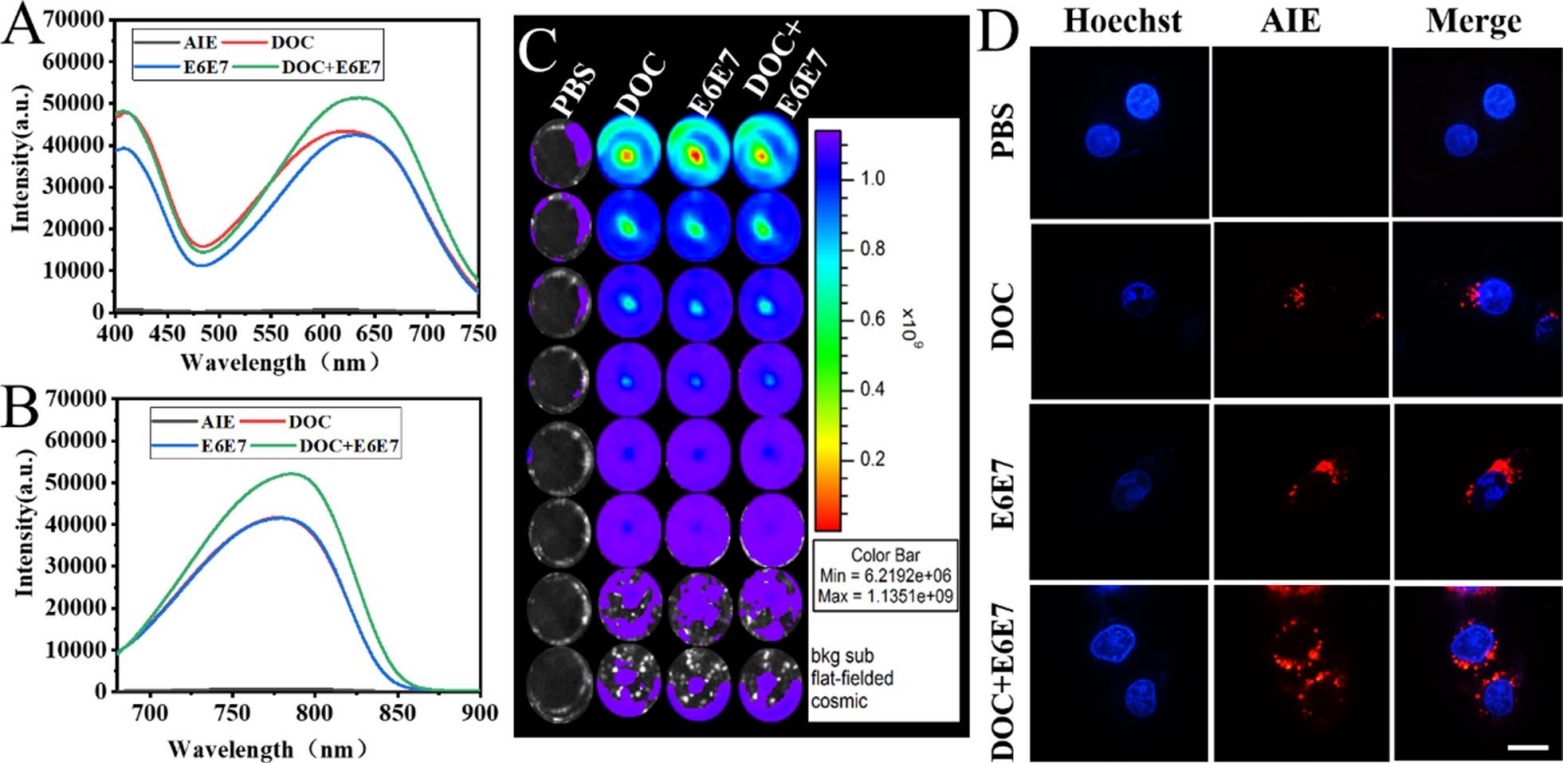
Optical performance of the (DOC + E6E7) @DOTAP nanoparticles. **A** Fluorescence excitation spectra. **B** Fluorescence emission spectra. **C** Fluorescence properties of different samples after AIE was embedded in the hydrophobic shell of DOTAP were monitored by an in vivo imaging system (IVIS). **D** Confocal laser scanning microscope images of Hela cells incubated with nanoparticles for 6 h. Blue and red fluorescence represent Hoechst-stained nuclei and AIE fluorescence, respectively. Scale bars, 20 μm

In order to track the position of the nanosystem in the body in real time, we embedded the AIE fluorophore from the TQ-BPN molecule in the nanosystem. The optical properties of the AIE from the TQ-BPN molecule, embedded in the hydrophobic shell of the amphiphilic DOTAP from (DOC + E6E7) @DOTAP nanoparticles, were tested by fluorescence spectrometer, and in vivo luminescence imaging system. The results showed that the largest absorption peak was at 625 nm and the largest emission peak was at 810 nm for the nanoparticles (Fig. 4A, B). The nanoparticles were added to the 96-well plate with different concentrations, and fluorescence from TQ-BPN was monitored using an in vivo imaging system (IVIS) (Fig. 4C). The results showed that as the concentration of nanoparticles increased, the fluorescence intensity gradually increased, and the substances encapsulated in the nanoparticles did not affect the fluorescence signal of the TQ-BPN molecules. Subsequently, we verified the endocytosis efficiency of HeLa cells for nanoparticles. For cellular uptake experiment, the uptake of the nanoparticles by HeLa cells were visualized by fluorescence microscope (Fig. 4D). As seen in the fluorescence images, the blue fluorescence and red fluorescence represent Hoechst labeled nucleus and TQ-BPN in cells, respectively. As shown in Fig. 4D, the nanoparticles were effectively internalized by HeLa cells. Therefore, TQ-BPN molecules could be used as molecular probes for observing nanoparticle integrity to indicate the release of DOC and Cas9 plasmid in vivo extracellularly. The above results revealed that TQ-BPN was successfully coated in nanoparticles and endocytosed by HeLa cells to exert its tracking effect in vivo.

### Therapeutic effects in vitro

**Fig. 5:**
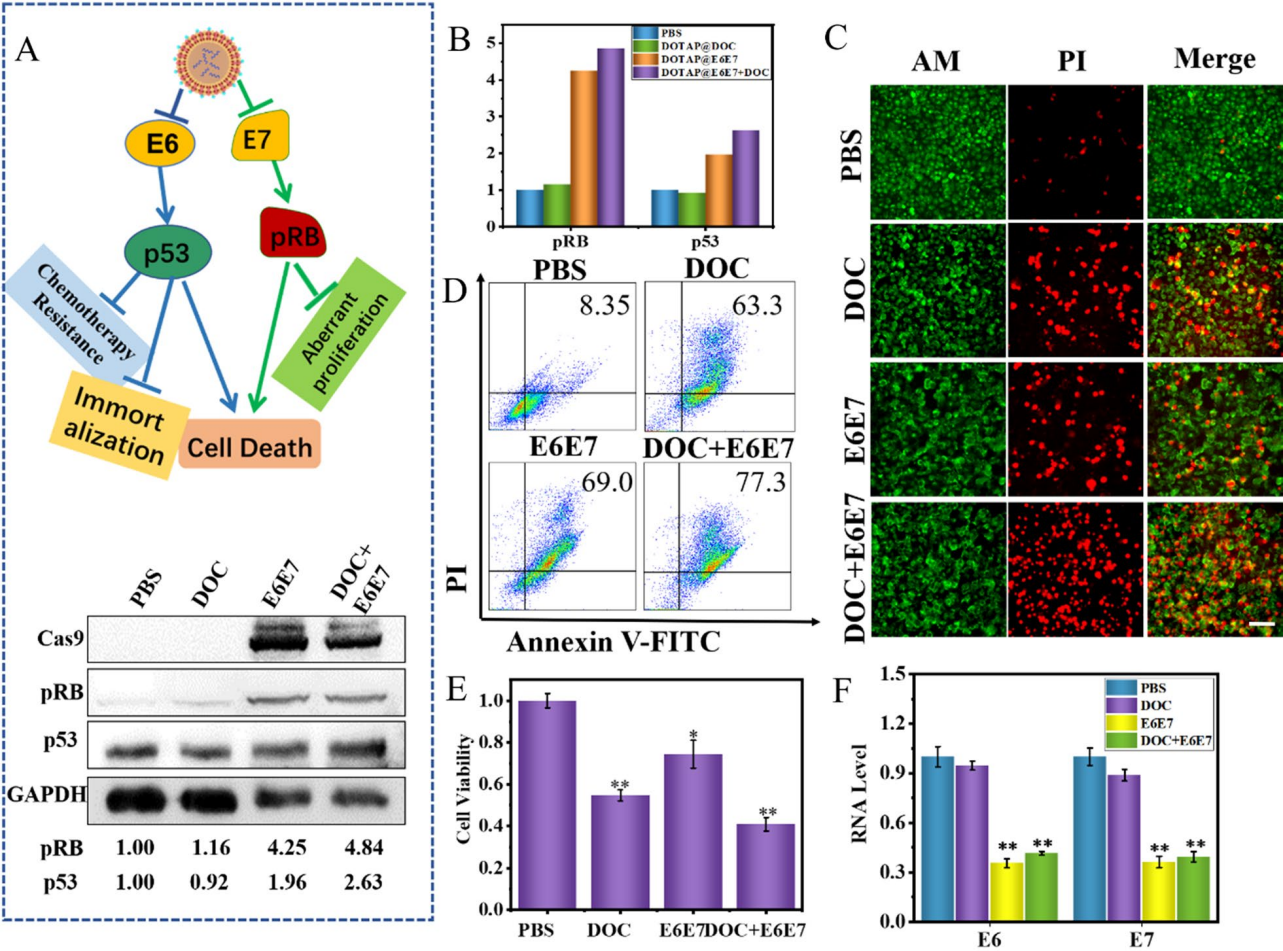
(DOC + E6E7) @DOTAP reduces cell viability and induces cell apoptosis. **A** HeLa (HPV18 positive) cells were treated with (DOC + E6E7) @DOTAP or control for 48 h before protein expression of various growth control proteins (p53 for E6 targeting, RB for E7 targeting) was determined by western blot analysis. **B** The relative density of bands was quantified using ImageJ software. **C**–**E** CCK-8 assay, cell apoptosis assay and Calcein-AM/PI staining. Cells were treated with the nanosystem for 48 h before cell apoptosis assay and Calcein-AM/PI staining. Scale bars, 200 μm. **F** E6 and E7 mRNA expression in the HeLa cells. *p < 0.05, **p < 0.01 vs. control

Before in vitro therapeutic experiments, the cytotoxicity of DOTAP and DOC was investigated via standard CCK-8 assays. After incubation with DOTAP at various concentrations for 24 h, negligible cytotoxicity was found even at a DOTAP concentration up to 100 µg/mL, indicating excellent cytocompatibility of DOTAP (Additional file 1: Fig. S6A, B). Binding ability of DOTAP to CRISPR/Cas9 vectors at different ratios demonstrated by the agarose gel retardation assay (Additional file 1: Fig. S6C). The results show that the binding ability is strongest when DNA: DOTAP is 1:2. In addition, the hydrodynamic particle size of (DOC + E6E7) @DOTAP remains stable at 24 and 48 h (Additional file 1: Fig. S6D). As the concentration of (DOC + E6E7) @DOTAP increases, the fluorescence excitation spectrum also increases (Additional file 1: Fig. S6E). Subsequently, the full spectrum of UV absorption of DOC was analyzed, and the results showed that DOC had a maximum absorption peak at 273 nm (Additional file 1: Fig. S7A). According to the absorption function of different concentrations of DOC at 273 nm, the encapsulation efficiency of DOC@DOTAP is calculated to be 56.2 % (Additional file 1: Fig. S7B). Next, we performed the western blotting assay at the same culture interval to verify whether the effect on HeLa cells was enhanced by enhancing p53 and pRB protein expression. The p53 and pRB levels in cells treated with the CRISPR/Cas9 carried by the nanoparticles were much higher than the mimic control group, indicating that the CRISPR/Cas9 maintained their function and affected HeLa cell apoptosis by gene regulation. Other control groups showed no significant up-regulation of p53 and pRB protein expression in HeLa cells (Fig. 5A, B). The cell apoptosis was examined by flow cytometry (FCM) which indicated that 84.1 % of the cells treated by the (DOC + E6E7) @DOTAP for 48 h underwent apoptosis (Fig. 5C). As a control, the DOC@DOTAP only led to 68 % cell apoptosis under the same conditions, indicating a specific gene knock-out of the E6 and E7. In addition, to directly visualize the therapeutic effects, cells under different treatments were incubated with calcein-AM/PI for dead (red) and live (green) cell staining. According to the confocal laser scanning microscope images (Fig. 5D), (DOC + E6E7) @DOTAP groups exhibited highest cellular mortality, which was consistent with the results of CCK-8 assays (Fig. 5E). As the concentration of (DOC + E6E7) @DOTAP nanoparticles increases, the number of apoptotic cells also increases, which proves that (DOC + E6E7) @DOTAP is directly proportional to the death rate of tumor cells (Additional file 1: Fig. S8A). In addition, the number of apoptotic cells increased with time after (DOC + E6E7) @DOTAP treated, which revealed that (DOC + E6E7) @DOTAP eliminates cervical cancer cells in a time and dose-dependent manner (Additional file 1: Fig. S8B, C). Furthermore, the (DOC + E6E7) @DOTAP nanoparticles decreased E6 and E7 mRNA expression, leading to p21 and p53 upregulation correspondingly, and significantly increases the mortality of cancer cells (Fig. 5F). These data prove that (DOC + E6E7) @DOTAP nanoparticles promote the death of cervical cancer cells and provide strong support for the further treatment of cervical cancer in vivo.

### In vivo therapeutic effects evaluation

**Fig. 6:**
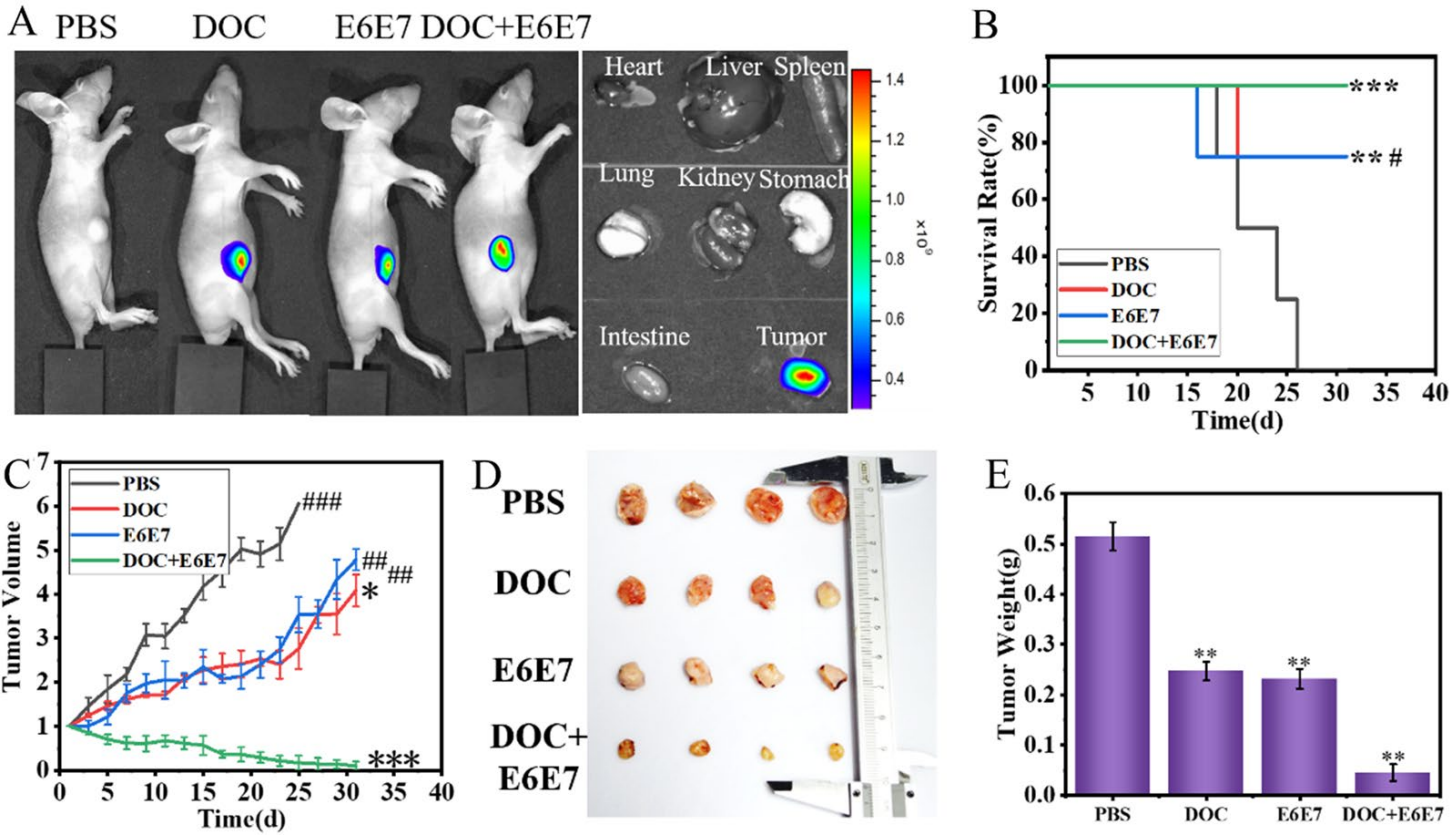
In vivo therapeutic effects for tumor in mice. **A** Representative fluorescence images using an in vivo imaging system (IVIS) depicting in vivo nanoparticles after intratumoral injection in different samples. HeLa cells were subcutaneously inoculated in mice and allowed to grow to 50 mm^3^ before being treated with the nanosystem. **B** The survival analysis of established HeLa xenografts in mice after treatment ((DOC + E6E7) @DOTAP), control (DOC@DOTAP, E6E7@DOTAP), or untreated (PBS only) in days. **C** Tumor volume was monitored for 31 days. **D** and **E** Size and weight of HeLa xenografts tumors for treating with different samples. *p < 0.05, **p < 0.01 vs. control. ^#^*p* < 0.05, ^##^*p* < 0.01, ^###^*p* < 0.05

We have verified the excellent efficacy of (DOC + E6E7) @DOTAP in killing cervical cancer cells in vitro, which encourages us to test the therapeutic effects of cervical cancer in vivo. In order to verify the therapeutic effect of nanoparticles in vivo, the in vivo combined therapeutic efficacy was evaluated on HeLa tumor-bearing mice. The study found that as-prepared (DOC + E6E7) @DOTAP were ideal nanomedicine with excellent hemocompatibility, which could be transported with safely in the blood fluid (Additional file 1: Fig. S9). After TQ-BPN molecules were embedded in the hydrophobic layer of nanoparticles, strong fluorescence could be observed in the tumor site by IVIS (Fig. 6A). After euthanasia, the tumors were isolated and dissected, so that a large amount of red AIE fluorescence could be observed in the tumor tissue. Here, the group 1 only accepted PBS intratumoral injection, group 2 only received DOC@DOTAP intratumoral injection, group 3 received E6E7@DOTAP intratumoral injected only, and group 4 received the (DOC + E6E7) @DOTAP intratumoral injection. The significant survival time was recorded for each group, suggested that the group of injection (DOC + E6E7) @DOTAP had a longer survival time than others, and the survival rate was 100 % in 30 days (Fig. 6B). As shown in Fig. 6C, the tumor of the mice injected by the DOC @DOTAP and E6E7 @DOTAP treated mice, showed tumor inhibition, with a tumor regrowth during the therapeutic process, suggesting that neither chemotherapy nor gene editing therapy alone can cure the tumor-bearing mice. Delightedly, the group mice of injection (DOC + E6E7) @DOTAP showed very low tumor growth, which demonstrated the outstanding combined therapeutic effects of (DOC + E6E7) @DOTAP (Additional file 1: Fig. S10A). Besides, each group of tumors in vitro photos were presented and displayed, and the tumor size and weight of the group with injection (DOC + E6E7) @DOTAP was a minimum, indicating that the (DOC + E6E7) @DOTAP group could effectively inhibit the growth of tumor, compared to other groups such as PBS, DOC @DOTAP and E6E7@DOTAP (Fig. 6D, E). The above results indicated intratumoral injection strategies with high drug delivery efficiency and low systemic distribution based on nanoparticles can efficiently ablate local tumors.

**Fig. 7:**
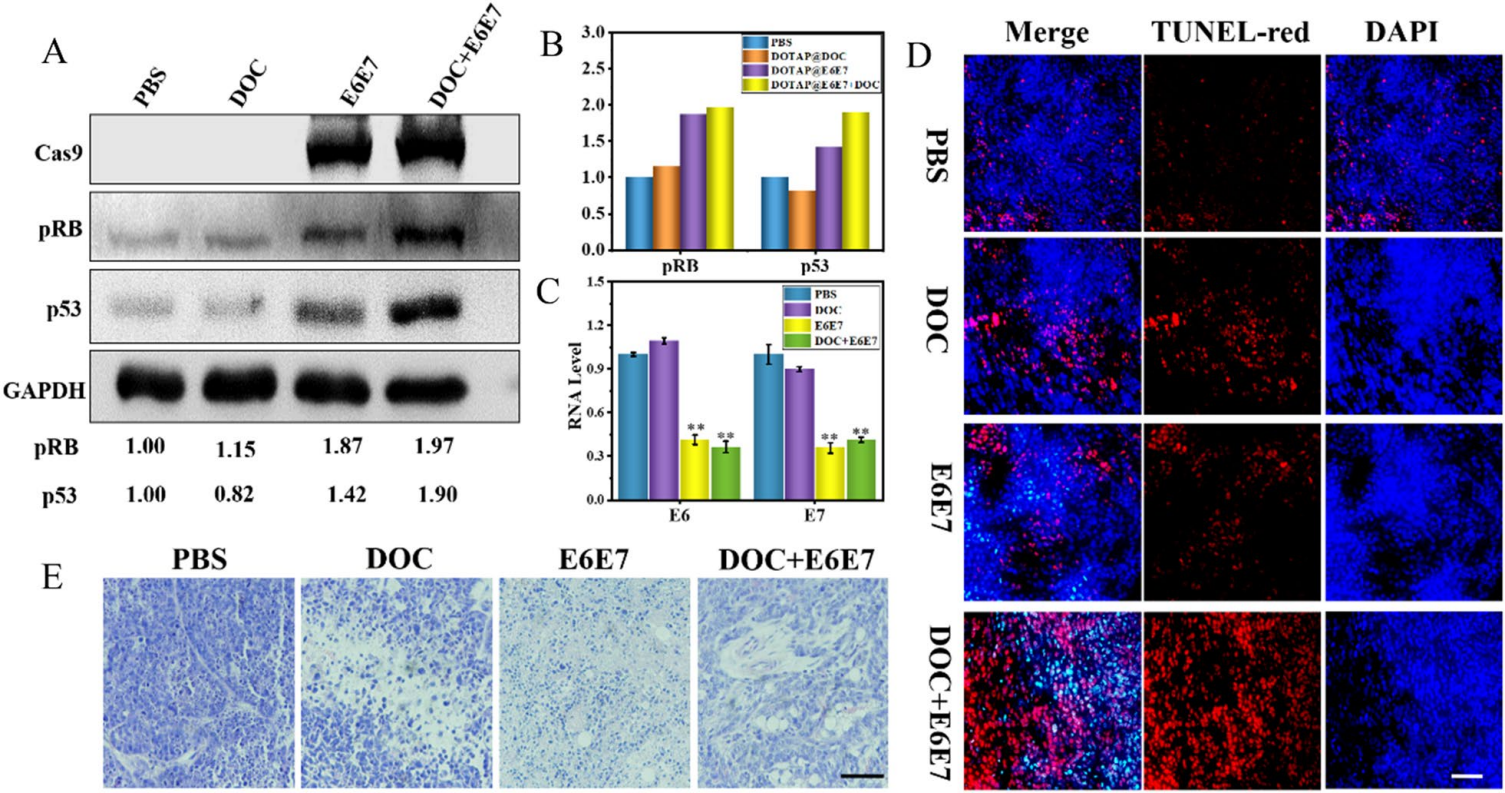
Induces cell apoptosis and key growth control proteins in vivo. **A** (DOC + E6E7) @DOTAP induces key growth control proteins (p53 for E6 targeting, RB for E7 targeting) in vivo. **B** Quantitative analysis of (**A**). HeLa cells were subcutaneously inoculated in mice and allowed to grow to 50 mm^3^ before being treated with the nanosystem. Subcutaneous injection with (DOC + E6E7) @DOTAP or control for 48 h before protein expression of various growth control proteins (p53 for E6 targeting, RB for E7 targeting) was determined by western blot analysis. The relative density of bands was quantified using ImageJ software. **C** E6 and E7 mRNA expression in subcutaneous. *p < 0.05, **p < 0.01 vs. control. **D** Immunohistochemical staining of tumor tissues with H&E. Scale bars, 200 μm. **E** TUNEL staining of tumor sections. Scale bars, 200 μm

Because (DOC + E6E7) @DOTAP nanoparticles have excellent cervical cancer treatment effects, we further evaluated the efficacy of nanoparticles targeting in vivo and possible mechanisms of action. Analysis of pRB and p53 protein expression of each tumor mass by Western Blot (Fig. 7A). The p53 and pRB level in groups treated with the E6E7 @DOTAP and (DOC + E6E7) @DOTAP was much higher than the PBS group. Quantitative analysis showed that compared with the PBS group, the expression of pRB protein increased by 1.87-fold and 1.97-fold, and the expression of p53 protein was increased by 1.42-fold and 1.90-fold, which mean that the antitumor effect was related to the p53 and pRB pathway (Fig. 7B). Quantitative RNA analysis showed the expression levels of E6 and E7 (Fig. 7C). In conclusion, the (DOC + E6E7) @DOTAP nanoparticles we prepared can specifically target cervical cancer and increase the therapeutic effect of the chemotherapy drug DOC by up-regulating the expression of p53 and pRB.

Next, we performed the terminal dexynucleotidyl transferase (TdT)-mediated dUTP nick end labeling (TUNEL) assay for detection of apoptosis of tumor cells. The treatment by (DOC + E6E7) @DOTAP significantly induced the TUNEL-positive tumor cells (red) in the dissected tumor tissues compared to other groups (Fig. 7D). We confirmed that the tumor inhibition was caused by (DOC + E6E7) @DOTAP induced tumor cell apoptosis by TUNEL assay. In addition, the HeLa xenograft tumors were collected and stained with hematoxylin and eosin. In PBS treatment group, poorly differentiated histology and the dense fibrotic tissue were discovered, and no vasculature could be observed inside tumor cell nests. However, the tumor cells in (DOC + E6E7) @DOTAP group exhibited obvious structural destruction and necrosis (Fig. 7E). In the meantime, the major organs of mice treated were collected for histological staining to compare with the control groups. No appreciable abnormality was observed in heart, liver, spleen, lung, and kidneys. In the process of all animal experiments, the weight of mice was recorded and suggested no obvious body weight fluctuation of mice and cell damage of main organs were found in different treatment groups (Additional file 1: Figs. S10B and S11). These results reveal that (DOC + E6E7) @DOTAP nanoparticles enhance the effect of chemotherapy drugs in the treatment of cervical cancer and significantly reduce the tolerance of cervical cancer cells to DOC, and (DOC + E6E7) @DOTAP nanoparticles have higher biocompatibility.

## Conclusions

To sum up, in order to overcome the challenge of cancer resistance, we design a CRISPR/Cas9 vector system that can delete a total of 563 bp of HPV18 E6 and E7 genes, effectively eliminating viral genes integrated into the host genome, simultaneously reactivate the p53 and pRB tumor suppressor pathways, resulting in cervical cancer cell death. In order to track the precise location of the nanoeditor in vivo in real time, a fluorescent molecule of TQ-BPN, owning to the aggregation-induced emission (AIE) effect in the infrared region, was selectively embedded in the hydrophobic shell of the amphiphilic cationic liposome. Excellent combined therapeutic efficacy and ignored side effects were observed in both in vitro and in vivo experiments, demonstrating the high superiority of the nanosystem. Combination therapy can reduce the amount of docetaxel (DOC) application, while the CRISPR/Cas9 system’s targeting can reduce side effects on other organs. DOC effect is much faster than CRISPR/Cas9 during their intracellular uptake. Therefore, the combination of CRISPR/Cas9-based therapy with traditional chemotherapy would be beneficial, which the challenge of tumor resistance has been effectively addressed. This facile yet versatile approach is also extremely easy to implement in other cancer treatments to customize personalized anti-tumor strategies by simply changing chemotherapy drugs and targeted genes.

## Supplementary Information

**Additional file 1:  Fig. S1.** CRISPR/Cas9 Plasmid Constructs and sgRNA Design.(A) Two pairs of sgRNAs were designedby using the ZiFit Web application (http://zifit.partners.org/) to target theamino-terminal regions of the HPV-18 E6 and E7 open reading frames (ORFs).(B) The secondary structures oftarget-sgRNAs. The secondarystructures were analyzed using the program RNA Folding Form (http://mfold.rna.albany.edu/?q=mfold/RNAFolding Form2.3). (C) PCRverification of CRISPR/Cas9 Plasmid Constructs. **Fig. S2.** Examples of direct sequencing of PCR products containing targeted sites. **Fig. S3.** Dual sgRNA-guided deletion of the HPV E6 and E7 Genes. (A) PCR amplification of the targetedregion. (B) T7E1 assay toanalyze the gene editing efficiency. **Fig. S4. **CRISPR/Cas9 PlasmidConstructs and sgRNA Design. Live anddead cells staining by Calcein-AM/PI for different cell line in vitro. Cells(293T; RD; A549; HEPG2; CasKi, HPV16 positive; 4T1) were treated withtarget-specific gRNA (E6E7) or control gRNA (non-specific), for 48 h beforestaining. Scale bars, 200 μm. **Fig. S5. **TEM image of (DOC+E6E7) @DOTAP. **Fig. S6.** The Process ofSynthesis of DOTAP@(DOC+E6E7) nanoparticles. (A) Relative viability of Hela cells cocultured with DOTAP atdifferent concentrations for 24 h. (B)Relative viability of Hela cells cocultured with DOC at differentconcentrations for 24 h. (C) Binding ability of DOTAP to CRISPR/Cas9 vectors atdifferent ratios demonstrated by the agarose gel retardation assay. (D) DLScharacterization of (DOC+E6E7) @DOTAP at different time points. (E)Fluorescence excitation spectra of different concentrations of (DOC+E6E7)@DOTAP. **Fig. S7. **(A) UV absorption curve of DOC. B) DOCconcentration-ultraviolet absorbance correlation curve. **Fig. S8. **Thetherapeutic efficiency of DOTAP@(DOC+E6E7) is dose-dependent (A) andtime-dependent (B, C). **Fig. S9. **Hemolytic property ofnanoparticles with mouse red blood cells. Scalebars, 200 μm. **Fig. S10.** (A)Photographs of tumor changes in HeLa-bearing mice after different sampletreatments. (B) The weight change in theHela subcutaneous tumor model in mice for treating with different ways. **Fig. S11.** H&E staining images ofmajor organs obtained from mice in PBS and (DOC+E6E7) @ DOTAP groups. Scale bars, 200 μm.

## Data Availability

All data about this study are included in this published article and its additional file.
