# Supplementary material for: CRISPR/Cas9 nanoeditor of double knockout large fragments of E6 and E7 oncogenes for reversing drugs resistance in cervical cancer
Source: J Nanobiotechnology. 2021 Aug 5;19:231. doi: 10.1186/s12951-021-00970-w (PMC8340365; doi:10.1186/s12951-021-00970-w)
Supplement: Supplementary file 1 — Additional file 1: Fig. S1. CRISPR/Cas9 Plasmid Constructs and sgRNA Design.(A) Two pairs of sgRNAs were designedby using the ZiFit Web application (http://zifit.partners.org/) to target theamino-terminal regions of the HPV-18 E6 and E7 open reading frames (ORFs).(B) The secondary structures oftarget-sgRNAs. The secondarystructures were analyzed using the program RNA Folding Form (http://mfold.rna.albany.edu/?q=mfold/RNAFolding Form2.3). (C) PCRverification of CRISPR/Cas9 Plasmid Constructs. Fig. S2. Examples of direct sequencing of PCR products containing targeted sites. Fig. S3. Dual sgRNA-guided deletion of the HPV E6 and E7 Genes. (A) PCR amplification of the targetedregion. (B) T7E1 assay toanalyze the gene editing efficiency. Fig. S4. CRISPR/Cas9 PlasmidConstructs and sgRNA Design. Live anddead cells staining by Calcein-AM/PI for different cell line in vitro. Cells(293T; RD; A549; HEPG2; CasKi, HPV16 positive; 4T1) were treated withtarget-specific gRNA (E6E7) or control gRNA (non-specific), for 48 h beforestaining. Scale bars, 200 μm. Fig. S5. TEM image of (DOC+E6E7) @DOTAP. Fig. S6. The Process ofSynthesis of DOTAP@(DOC+E6E7) nanoparticles. (A) Relative viability of Hela cells cocultured with DOTAP atdifferent concentrations for 24 h. (B)Relative viability of Hela cells cocultured with DOC at differentconcentrations for 24 h. (C) Binding ability of DOTAP to CRISPR/Cas9 vectors atdifferent ratios demonstrated by the agarose gel retardation assay. (D) DLScharacterization of (DOC+E6E7) @DOTAP at different time points. (E)Fluorescence excitation spectra of different concentrations of (DOC+E6E7)@DOTAP. Fig. S7. (A) UV absorption curve of DOC. B) DOCconcentration-ultraviolet absorbance correlation curve. Fig. S8. Thetherapeutic efficiency of DOTAP@(DOC+E6E7) is dose-dependent (A) andtime-dependent (B, C). Fig. S9. Hemolytic property ofnanoparticles with mouse red blood cells. Scalebars, 200 μm. Fig. S10. (A)Photographs of tumor changes in HeLa-bearing mice after di [file 12951_2021_970_MOESM1_ESM.docx]

**Additional Material**

**CRISPR/Cas9 nanoeditor of double knockout large fragments of E6 and E7 oncogenes for reversing drugs resistance in cervical cancer**

Xianhuang Li, ^1, †^ Mingming Guo, ^2, †^ Bei Hou, ^1, †^ Bin Zheng ^2,^ * Zhiyun Wang, ^3^ Mengqian Huang, ^1^ Yanan Xu, ^1^ Jin Chang ^1^ and Tao Wang ^1, *^

^1^ School of Life Sciences, Tianjin University, 92 Weijin Road, Nankai District, Tianjin 300072, China.

^2^ Academy of Medical Engineering and Translational Medicine, Tianjin Key Laboratory of Brain Science and Neural Engineering, Tianjin University, 92 Weijin Road, Nankai District, Tianjin 300072, China.

^3^ School of Environmental Science and Engineering, Tianjin University, 92 Weijin Road, Nankai District, Tianjin 300072, China.

E-mail addresses: wangtaobio@tju.edu.cn (T. Wang), binzheng@tju.edu.cn (B. Zheng).

^†^ These authors contributed equally to this work.


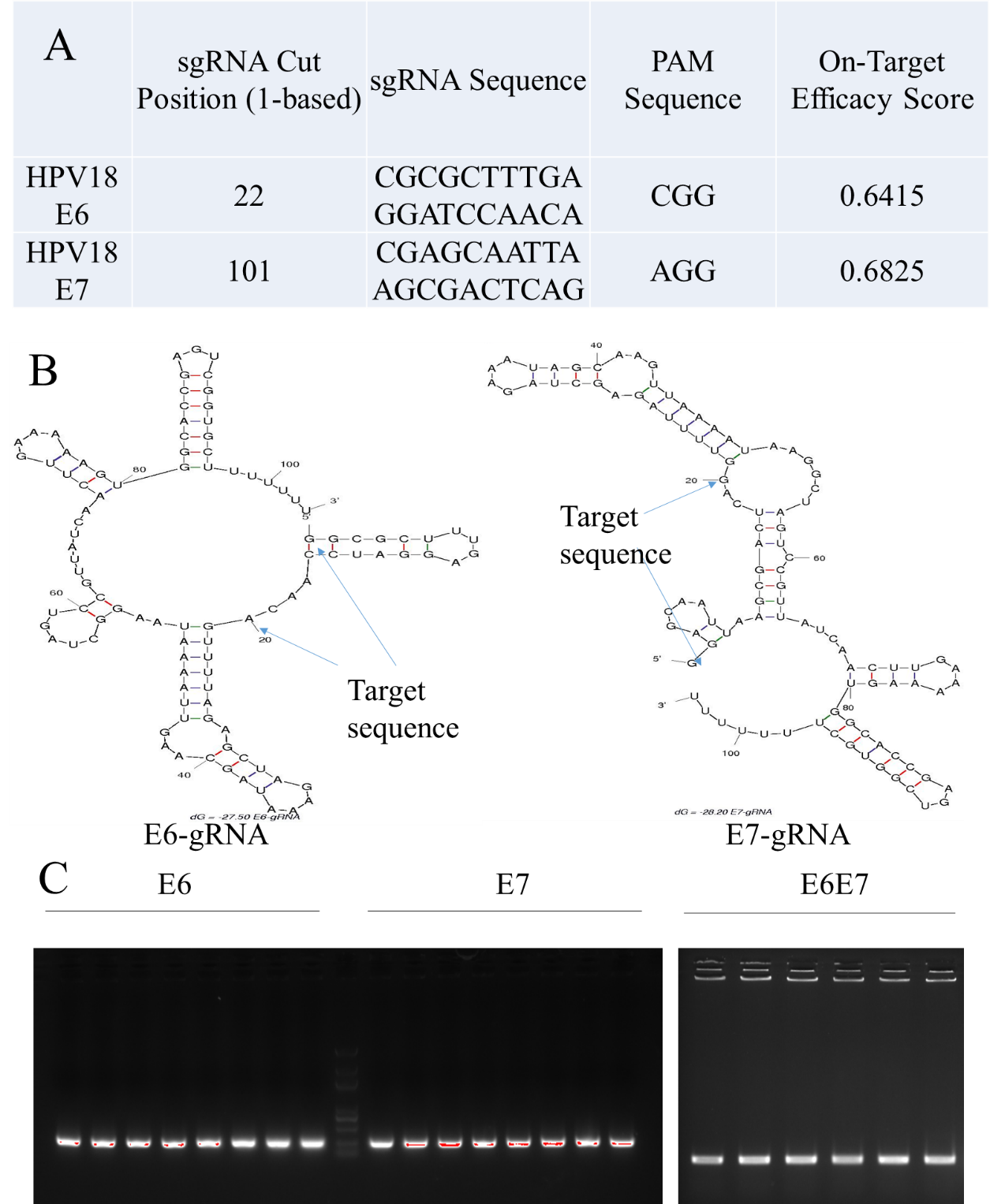


**Fig. S1.** CRISPR/Cas9 Plasmid Constructs and sgRNA Design. (A) Two pairs of sgRNAs were designed by using the ZiFit Web application (http://zifit.partners.org/) to target the amino-terminal regions of the HPV-18 E6 and E7 open reading frames (ORFs). (B) The secondary structures of target-sgRNAs. The secondary structures were analyzed using the program RNA Folding Form (http://mfold.rna.albany.edu/?q=mfold/RNA Folding Form2.3). (C) PCR verification of CRISPR/Cas9 Plasmid Constructs.


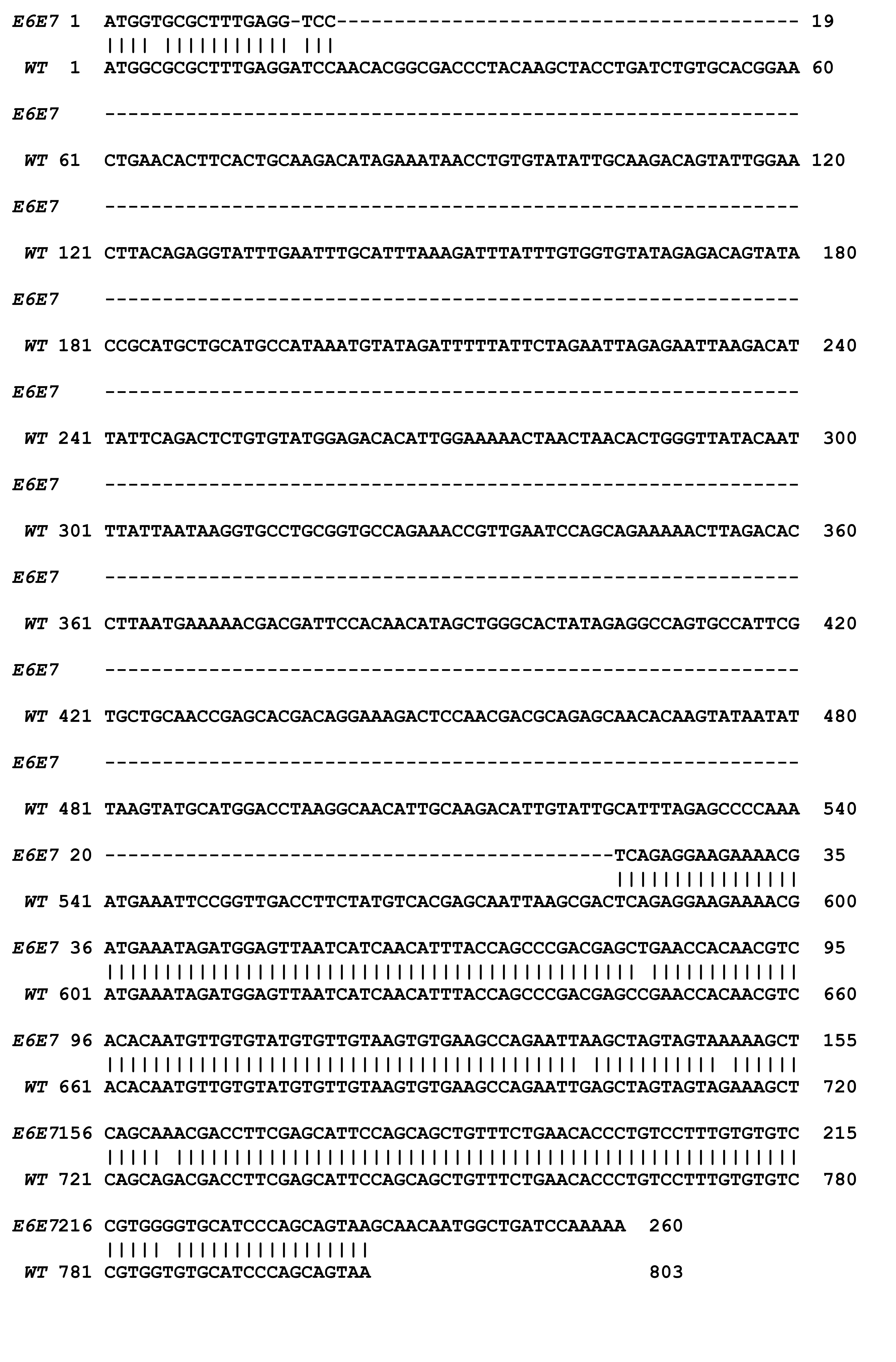


**Fig. S2.** Examples of direct sequencing of PCR products containing targeted sites.


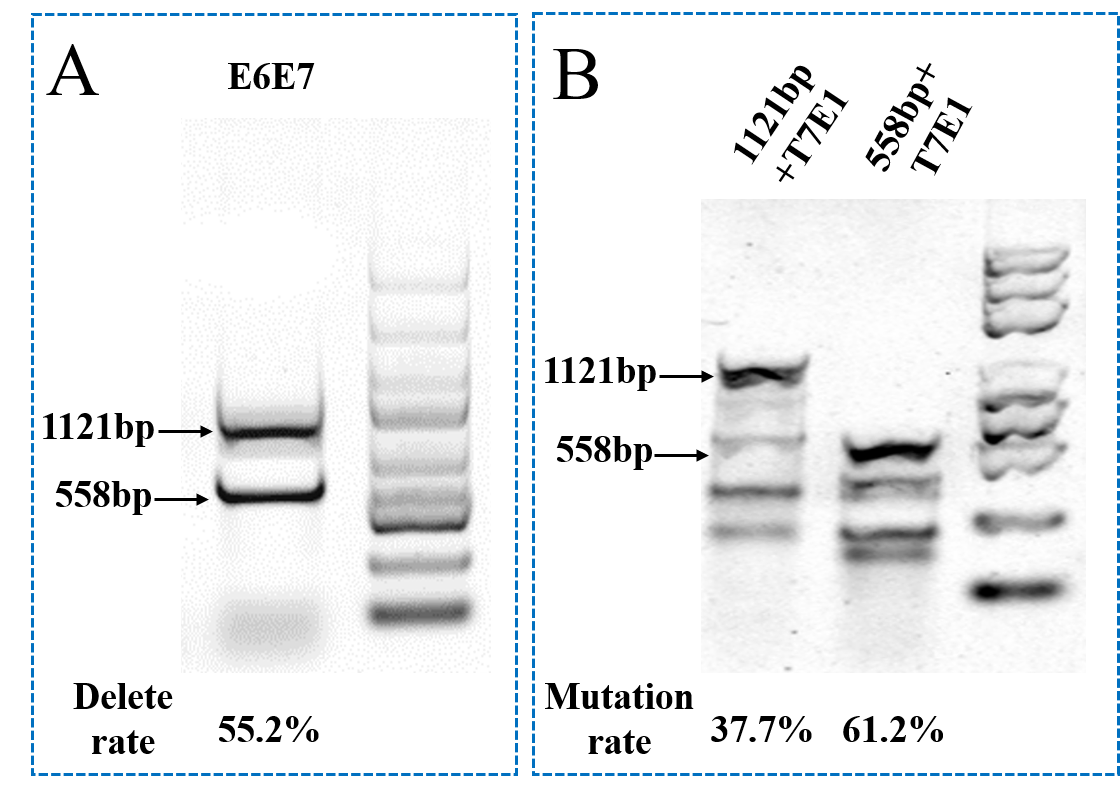


**Fig. S3.** Dual sgRNA-guided deletion of the HPV E6 and E7 Genes. (A) PCR amplification of the targeted region. (B) T7E1 assay to analyze the gene editing efficiency.


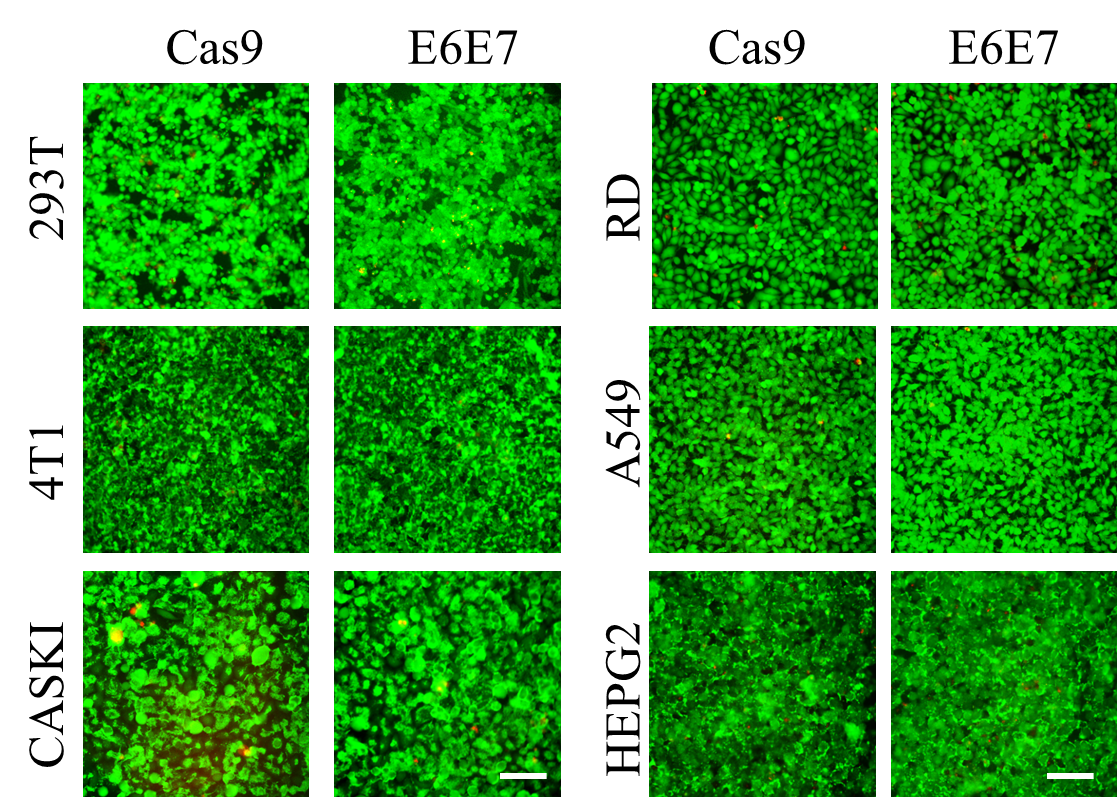


**Fig. S4.** CRISPR/Cas9 Plasmid Constructs and sgRNA Design. Live and dead cells staining by Calcein-AM/PI for different cell line in vitro. Cells (293T; RD; A549; HEPG2; CasKi, HPV16 positive; 4T1) were treated with target-specific gRNA (E6E7) or control gRNA (non-specific), for 48 h before staining. Scale bars, 200 μm.


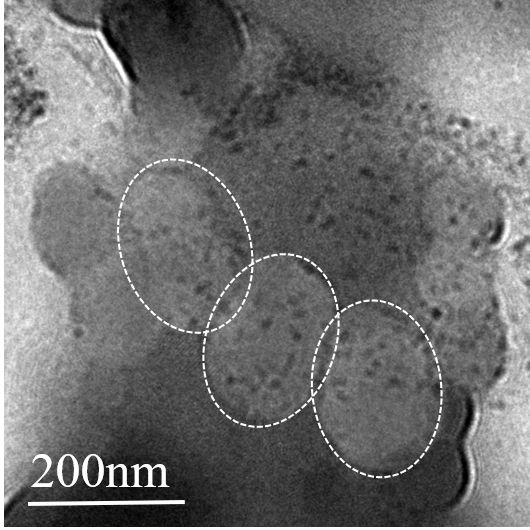


**Fig. S5.** TEM image of (DOC+E6E7) @DOTAP.


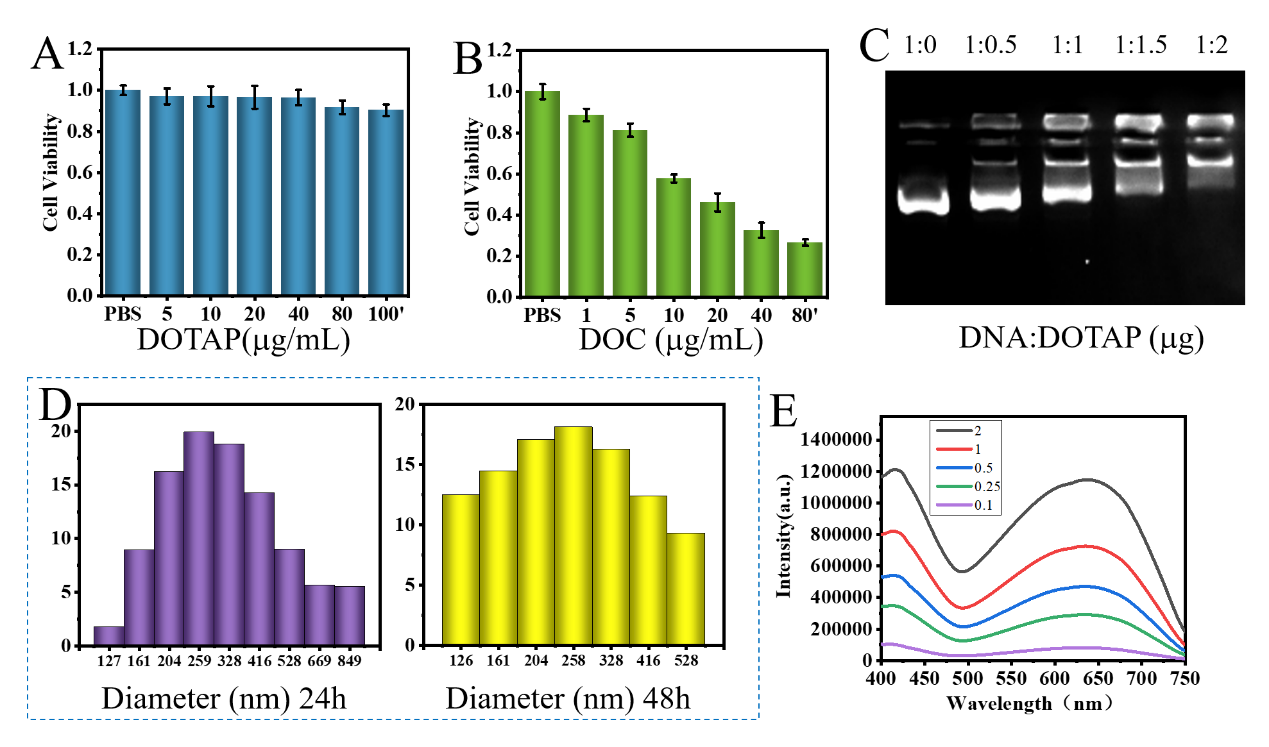


**Fig. S6.** The Process of Synthesis of DOTAP@(DOC+E6E7) nanoparticles. (A) Relative viability of Hela cells cocultured with DOTAP at different concentrations for 24 h. (B) Relative viability of Hela cells cocultured with DOC at different concentrations for 24 h. (C) Binding ability of DOTAP to CRISPR/Cas9 vectors at different ratios demonstrated by the agarose gel retardation assay. (D) DLS characterization of (DOC+E6E7) @DOTAP at different time points. (E) Fluorescence excitation spectra of different concentrations of (DOC+E6E7) @DOTAP.


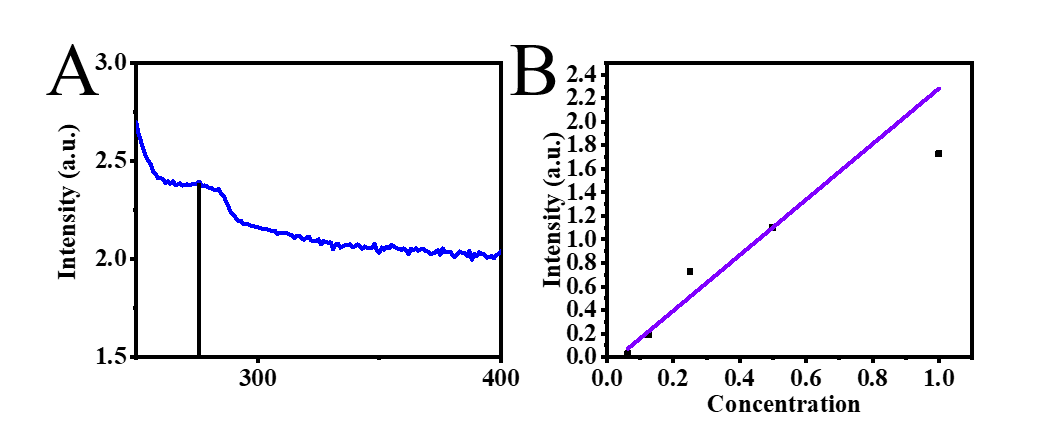


**Fig. S7.** (A) UV absorption curve of DOC. B) DOC concentration-ultraviolet absorbance correlation curve.


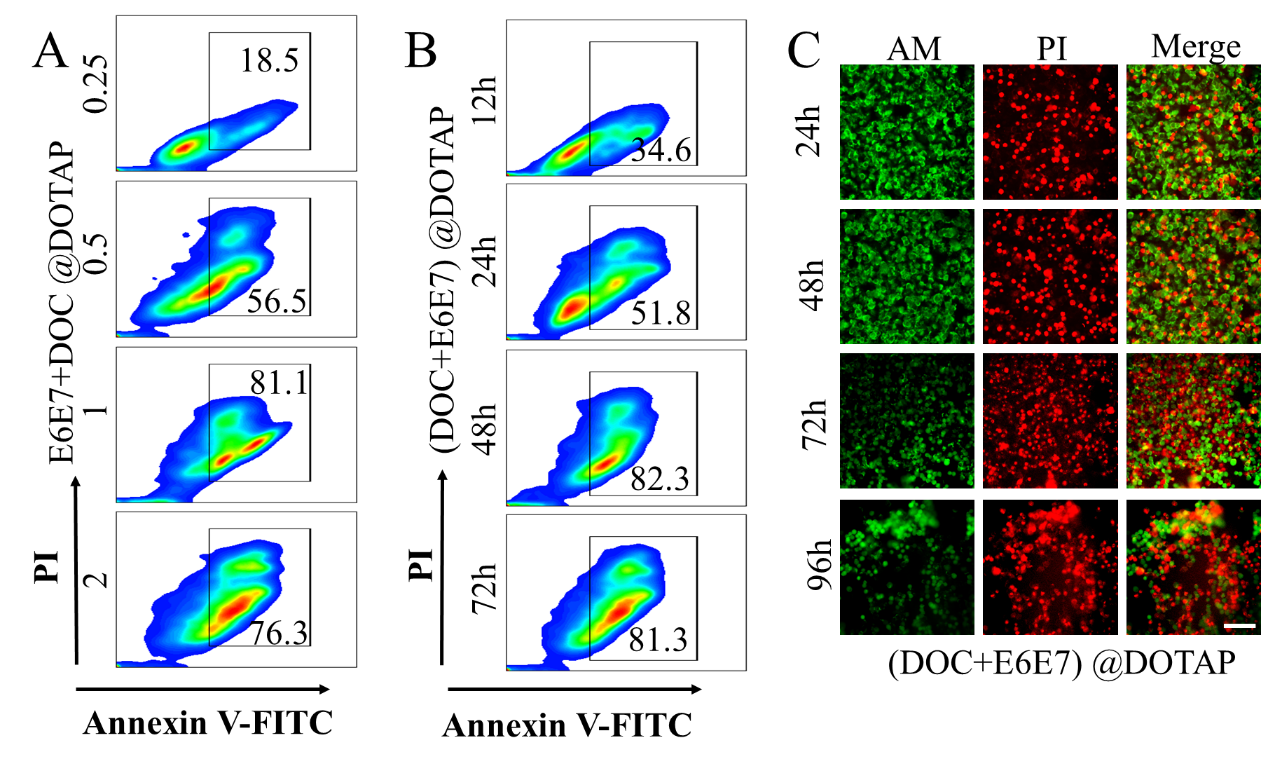


**Fig. S8.** The therapeutic efficiency of DOTAP@(DOC+E6E7) is dose-dependent (A) and time-dependent (B, C).


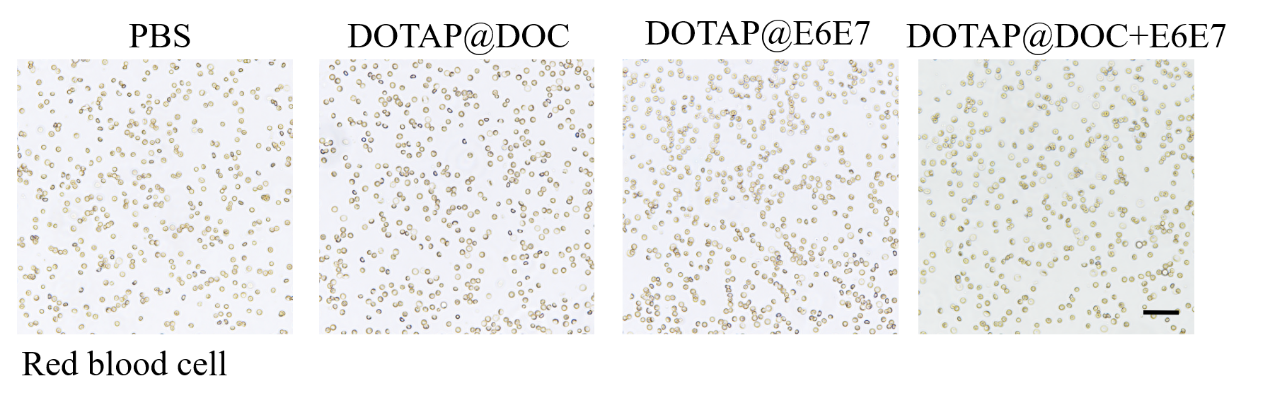


**Fig. S9.** Hemolytic property of nanoparticles with mouse red blood cells. Scale bars, 200 μm.


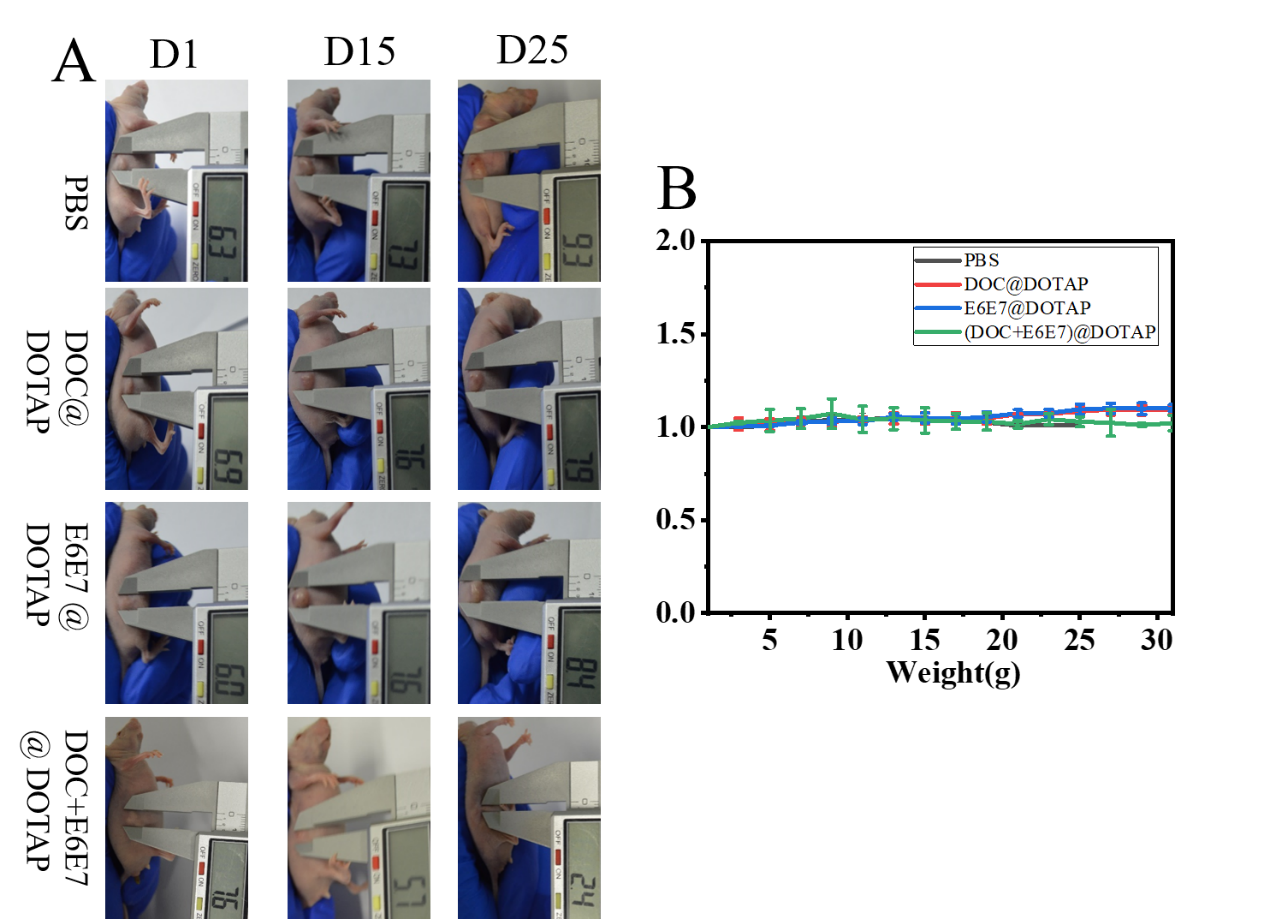


**Fig. S10.** (A) Photographs of tumor changes in HeLa-bearing mice after different sample treatments. (B) The weight change in the Hela subcutaneous tumor model in mice for treating with different ways.


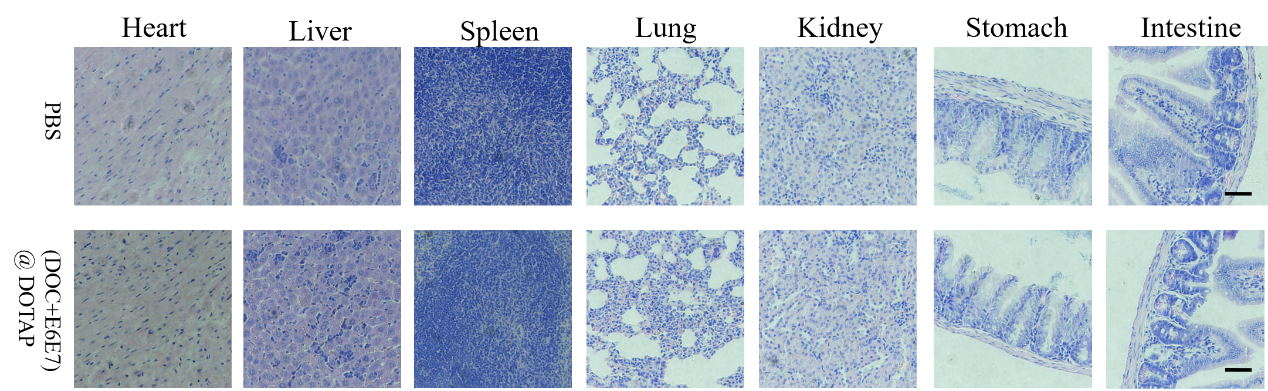


**Fig. S11.** H&E staining images of major organs obtained from mice in PBS and (DOC+E6E7) @ DOTAP groups. Scale bars, 200 μm
